# Sport-based interventions for post-disaster mental health and psychosocial recovery: a multi-level systematic review and meta-analysis within the MHPSS framework

**DOI:** 10.3389/fpubh.2026.1865744

**Published:** 2026-09-15

**Authors:** Tayfun Kara

**Affiliations:** Department of Sport Management, Faculty of Sport Sciences, Kahramanmaraş Sütçü İmam University, Kahramanmaraş, Türkiye

**Keywords:** community resilience, physical activity, post-disaster psychosocial support, post-traumatic stress disorder, sport-based intervention

## Abstract

Disasters and humanitarian crises affect both individual mental health and broader psychosocial functioning, but these outcome domains are not interchangeable. This systematic review and meta-analysis examined structured sport-, exercise-, play-, yoga/body-awareness-, physical-education-, and sport-for-protection-based interventions within a mental health and psychosocial support (MHPSS) framework. Web of Science Core Collection, PubMed, Scopus, SPORTDiscus, PsycINFO, and Google Scholar were searched for studies published from January 1, 2000, through November 1, 2025. Eligible populations were affected by disasters, conflict, forced displacement, refugeehood, humanitarian crises, or crisis-related restrictions. Outcome-specific quantitative analyses were conducted for comparable mental health symptoms, whereas heterogeneous psychosocial and community constructs were synthesized narratively and integrated through a joint interpretation matrix. Twenty-eight studies or programme evaluations were included; 20 contributed data to at least one quantitative analysis, with fewer studies contributing to each controlled outcome model. Random-effects models indicated favorable small-to-moderate average effects for PTSD symptoms (Hedges’ *g* = −0.32, 95% CI [−0.50, −0.14]; *I*^2^ = 58%), depression (*g* = −0.44, 95% CI [−0.60, −0.28]; *I*^2^ = 47%), and anxiety (*g* = −0.41, 95% CI [−0.58, −0.24]; *I*^2^ = 52%). Narrative findings identified routine, emotional regulation, belonging, social connectedness, life skills, and community cohesion as possible complementary pathways and outcomes. However, variation in populations, designs, intervention components, comparators, and measures, together with risk-of-bias concerns and small outcome sets, limits certainty and generalizability. Sport-based interventions may function as scalable community or focused non-specialized components of MHPSS when they are trauma-sensitive, culturally adapted, safeguarded, monitored, and linked to referral pathways. They should not be interpreted as substitutes for specialized mental health care, trauma-focused treatment, child protection, or clinical risk management.

## 1 Introduction

Disasters are multidimensional crises that profoundly affect individuals’ mental health, social relationships, sense of security, and communities’ capacity for reorganization (1). Extraordinary situations such as earthquakes, floods, epidemics, war, and mass displacement interrupt individuals’ living spaces, social support networks, economic security, and expectations regarding the future, thereby extending the effects of traumatic experience far beyond the moment of the event itself (2). Factors emerging after disasters, such as loss, uncertainty, housing problems, disruption of family integrity, and interruption of educational and working life, may pave the way for psychological outcomes such as post-traumatic stress disorder, depression, anxiety, sleep disorders, and a persistent state of hypervigilance (3–6).

In this review, “sport-based interventions” is an umbrella term for structured exercise, play, sport-for-protection, yoga or body-awareness practices, physical education, and planned physical activity delivered with an explicit mental health, psychosocial, protective, or recovery-oriented purpose. Two outcome families are distinguished throughout. Mental health outcomes refer to symptoms or clinically adjacent indicators assessed at the individual level, including post-traumatic stress, depression, anxiety, and suicidal ideation. Psychosocial outcomes refer to individual–social and community processes such as emotional regulation, perceived control, routine, social connectedness, belonging, life skills, social participation, community cohesion, and resilience. These domains may interact, but they are not treated as interchangeable: symptom change was eligible for outcome-specific quantitative pooling when comparable data were available, whereas heterogeneous psychosocial processes were synthesized narratively. This distinction follows the layered logic of mental health and psychosocial support (MHPSS), in which basic services and security, community and family supports, focused non-specialized supports, and specialized services are complementary rather than substitutable (7, 8).

Contemporary disaster management approaches acknowledge that psychosocial recovery is as critical as physical recovery (9). However, access to professional mental health services in post-disaster settings is often limited. The inadequacy of services, spatial and economic barriers, concerns about stigmatization, the collective nature of trauma, and individuals’ distant attitudes toward traditional psychological counselling services increase the need for complementary and community-based forms of intervention (10). At this point, physical activity, play, and sport-based interventions are regarded as holistic psychosocial support tools with the potential to strengthen emotional regulation, social reconnection, routine formation, and community solidarity. The proposed model is presented in Figure 1 as a conceptual framework linking sport-based interventions with biological, psychological, and social recovery pathways in post-disaster contexts.

**Figure 1:**
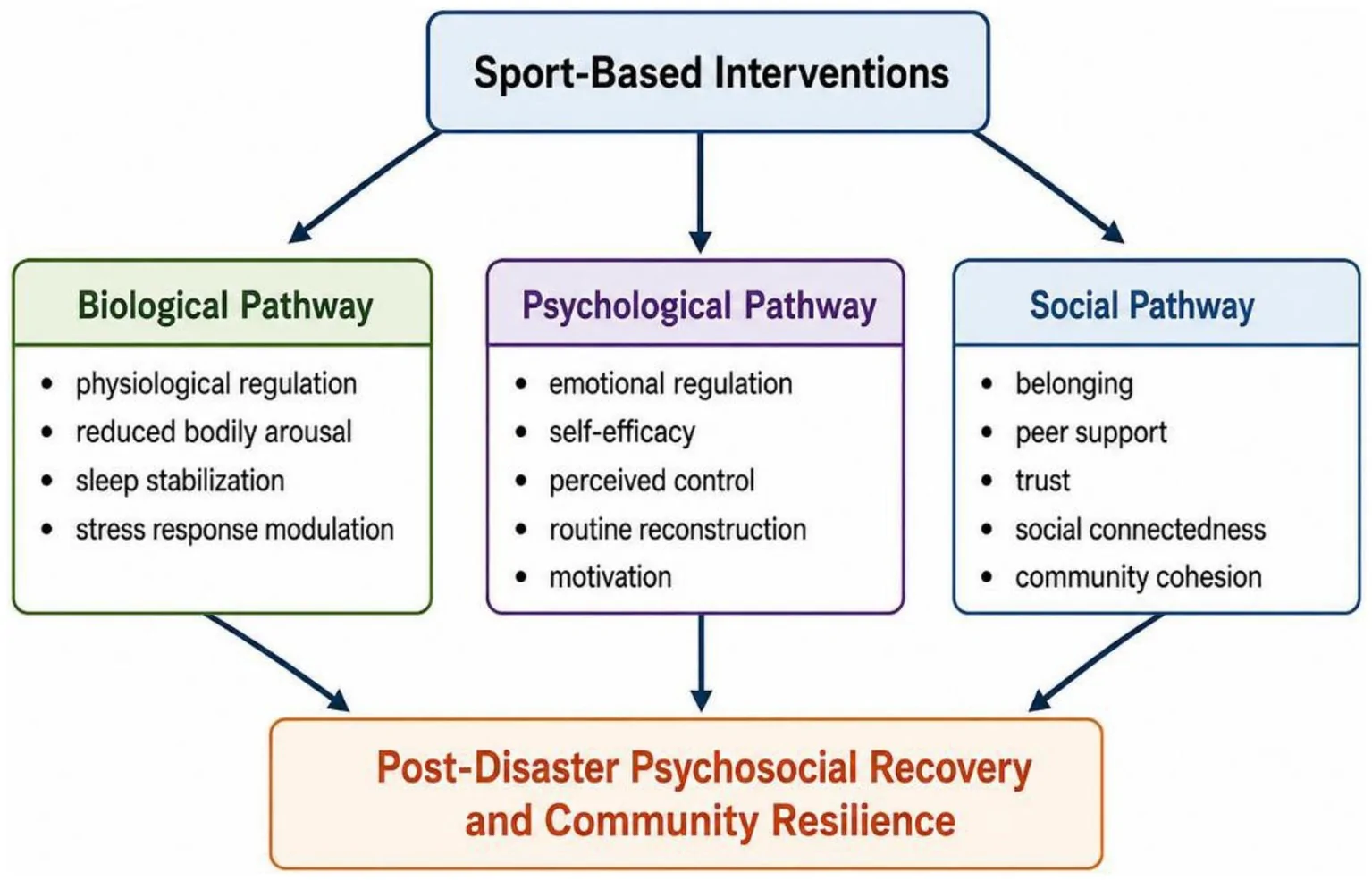
Sport-based psychosocial recovery model.

The importance of sport-based interventions in the context of post-disaster recovery stems from their capacity to affect individual and social levels simultaneously. Physical activity may generate regulatory effects on body perception, sleep patterns, energy levels, and mood, which are disrupted after trauma (11). Structured exercise, yoga, team games, and play-based sport programmes may help individuals re-establish bodily awareness. In addition, sport and play activities carried out in groups provide individuals with a safe space for social contact, strengthen peer support, create a sense of common purpose, and may reconstruct the sense of belonging weakened after traumatic experience (12). Therefore, sport-based programmes may offer a more natural, accessible, and easily participatory form of support for children and young people, particularly when compared with psychosocial interventions based on verbal expression (13–15).

Another important aspect of sport and physical activity in post-disaster conditions is their ability to generate routine and predictability in environments where everyday life has been interrupted. Regular physical activities enable individuals to acquire a structured rhythm during the day, reduce bodily tension, and engage in safe interaction with their social environment (16). Sport-based programmes implemented in temporary accommodation areas, schools, community centres, and youth spaces may also support the reorganization of social life after disasters. Elements such as teamwork, fair play, cooperation, mutual trust, and orientation toward a common goal make visible the community-level solidarity-strengthening dimension of sport (17). Within this framework, sport-based psychosocial support programmes conducted with children and adolescents carry particular importance. It may not always be possible for children to directly express, in verbal form, the feelings of fear, loss, insecurity, and uncertainty they experience after disasters (18). Play and sport may mediate the expression of these emotions in more indirect, safe, and developmentally appropriate ways. Movement, play, and group interaction may support the child in regaining a sense of control, forming bonds with peers, and feeling competent. For this reason, physical education, sport, and play activities may be considered among the complementary components of post-disaster child protection and psychosocial support systems (19–21). Another advantage of sport-based interventions is that they are relatively low-cost, culturally adaptable, and applicable on a community basis. In post-disaster environments where resources are limited, it may not always be possible to deliver individual therapy services requiring high levels of expertise to large population groups (22). In contrast, appropriately structured sport, play, and physical activity programmes can reach broader populations through the participation of teachers, coaches, volunteers, local institutions, and community leaders (23). However, this accessibility does not mean that every sport-based programme will automatically produce a therapeutic effect. Interventions must be designed in a trauma-sensitive manner, participant safety must be ensured, they must be carried out with content appropriate to age and developmental stage, and they must be adapted to the cultural context (24–26). To strengthen the theoretical basis of this review, the present study is framed around a multi-level Sport-Based Psychosocial Recovery Model. This model assumes that sport-based interventions may influence post-disaster recovery through three interrelated pathways (27). The first is a biological pathway, through which structured movement may contribute to physiological regulation, reduced bodily arousal, improved sleep, and stabilization of stress-related somatic responses (28). The second is a psychological pathway, through which sport and play may support self-efficacy, emotional regulation, perceived control, motivation, and the reconstruction of daily routine. The third is a social pathway, through which group-based activities may foster connectedness, belonging, peer support, trust, communication, and community cohesion (29). The 2023 Kahramanmaraş earthquakes illustrate the relevance of this multi-level perspective, as such disasters disrupt not only individual psychological functioning but also the routines, relationships, and community structures that sustain recovery (30).

Although the literature presents promising findings regarding post-disaster sport and physical activity, the evidence is fragmented across crisis contexts, intervention forms, populations, and outcome domains (12, 31, 32). Studies have examined earthquakes, war and displacement, refugee settings, disaster-risk communities, and pandemic restrictions; interventions range from yoga and structured exercise to play-based programmes, physical education, and sport-for-protection. Direct comparison is further complicated by small samples, short follow-up, inconsistent reporting of intervention fidelity, and substantial variation in outcome measures. The primary objective of this review was therefore to estimate outcome-specific effects on PTSD, depression, and anxiety when controlled quantitative data were sufficiently comparable. The secondary objective was to synthesize evidence concerning broader psychosocial recovery, including routine, emotional regulation, social support, belonging, life skills, community cohesion, and resilience. The review question was: among disaster-, displacement-, refugee-, pandemic-, war-, or humanitarian-crisis-affected populations, what effects do structured sport- and physical-activity-based interventions have on (a) individual mental health symptoms and (b) broader psychosocial recovery, compared with usual care, no intervention, waitlist, an alternative intervention, or—only in analyses kept separate from the main controlled models—baseline conditions?

## 2 Method

This systematic review and meta-analysis was designed to evaluate sport-based interventions across two related but analytically distinct evidence streams: outcome-specific quantitative evidence for mental health symptoms and structured narrative evidence for broader psychosocial recovery. The review question and eligibility criteria were specified using PICOS, and reporting followed PRISMA 2020 and Cochrane methodological guidance (33, 34).

### 2.1 Protocol and reporting framework

A review protocol was developed before full-text eligibility decisions, data extraction, and quantitative synthesis, but it was not prospectively registered in PROSPERO because preliminary scoping and screening had already begun when registration was considered. Before full-text assessment, the review team specified the review question, eligibility criteria, outcome families, extraction fields, risk-of-bias tools, and planned synthesis strategy. Because the protocol was not publicly time-stamped, selective methodological decision-making cannot be excluded completely; this limitation is considered when interpreting the review’s findings.

### 2.2 Information sources and search limits

Web of Science Core Collection, PubMed, Scopus, SPORTDiscus, PsycINFO, and Google Scholar were searched for records published from January 1, 2000, through November 1, 2025. The start date was selected to capture contemporary MHPSS practice and the period in which structured sport-, play-, movement-, and physical-activity-based programmes were increasingly reported in humanitarian and post-disaster settings. Search concepts covered three blocks—crisis exposure, sport or physical activity, and mental health or psychosocial outcomes—and were combined with Boolean operators. No language filter was applied during database searching. At full text, reports were included when they were available in English or Turkish or could be interpreted reliably by the review team; records that could not be accessed or interpreted sufficiently for eligibility assessment and extraction were excluded. Peer-reviewed studies and relevant official or programme reports were eligible, whereas popular media, opinion pieces without empirical or implementation data, and promotional materials were excluded (see Table 1).

**Table 1:** PICOS framework of the review.

PICOS component	Definition in this review
Population	Children, adolescents, adults, refugees, asylum seekers, displaced populations, and community groups affected by natural disasters, earthquakes, floods, pandemics, war/conflict, forced displacement, refugeehood, or humanitarian crises.
Intervention	Sport-based, exercise-based, play-based, physical activity, physical education, yoga/body-awareness, structured movement, and sport-for-protection interventions delivered with mental health, psychosocial, protective, or recovery-oriented aims.
Comparator	Usual care, no intervention, waitlist control, alternative psychosocial support, treatment as usual, or baseline/pre-intervention conditions where controlled data were not available.
Outcomes	PTSD symptoms, depression, anxiety, suicidal ideation, psychological resilience, quality of life, social support, social connectedness, life skills, psychosocial recovery, community cohesion, and community resilience.
Study designs	Randomized controlled trials, quasi-experimental studies, controlled intervention studies, cohort studies, mixed-methods studies, qualitative studies, field evaluations, and implementation reports. Only studies with sufficient quantitative outcome data were included in the meta-analysis.

### 2.3 Eligibility criteria, selection, and data extraction

Eligible populations were children, adolescents, or adults directly affected by a natural disaster, war or conflict, forced displacement, refugeehood, a humanitarian emergency, or crisis-related restrictions. Eligible interventions had to be structured, involve sport, exercise, play, physical activity, physical education, yoga/body awareness, or sport-for-protection, and state a mental health, psychosocial, protective, or recovery-oriented aim. For the systematic review, eligible reports had to provide empirical outcome data, qualitative findings, or implementation evidence relevant to at least one prespecified mental health or psychosocial domain. Protocols and publications based on overlapping samples could inform context or implementation but were not treated as independent outcome studies. Studies of routine physical activity without a crisis-exposed population, unstructured recreation without a stated recovery purpose, elite-sport performance, purely biomedical outcomes without a mental health or psychosocial measure, and reports lacking interpretable data were excluded. For the main meta-analysis, an independent comparison group and extractable post-intervention or compatible change-score data were additionally required; uncontrolled pre–post evidence was reserved for separate sensitivity or descriptive analyses.

Two reviewers independently screened titles and abstracts and then full texts. Disagreements were resolved by consensus; inter-rater agreement was strong (Cohen’s *κ* = 0.82). Extracted fields included publication year, country and crisis context, design, participant characteristics, sample size, intervention content and dose, comparator, measurement tool, follow-up, outcome data, implementation features, and synthesis role. When multiple reports used the same sample, the primary outcome report was used for pooled estimates and secondary reports were retained only for non-duplicative outcomes or contextual interpretation (see Figure 2).

**Figure 2:**
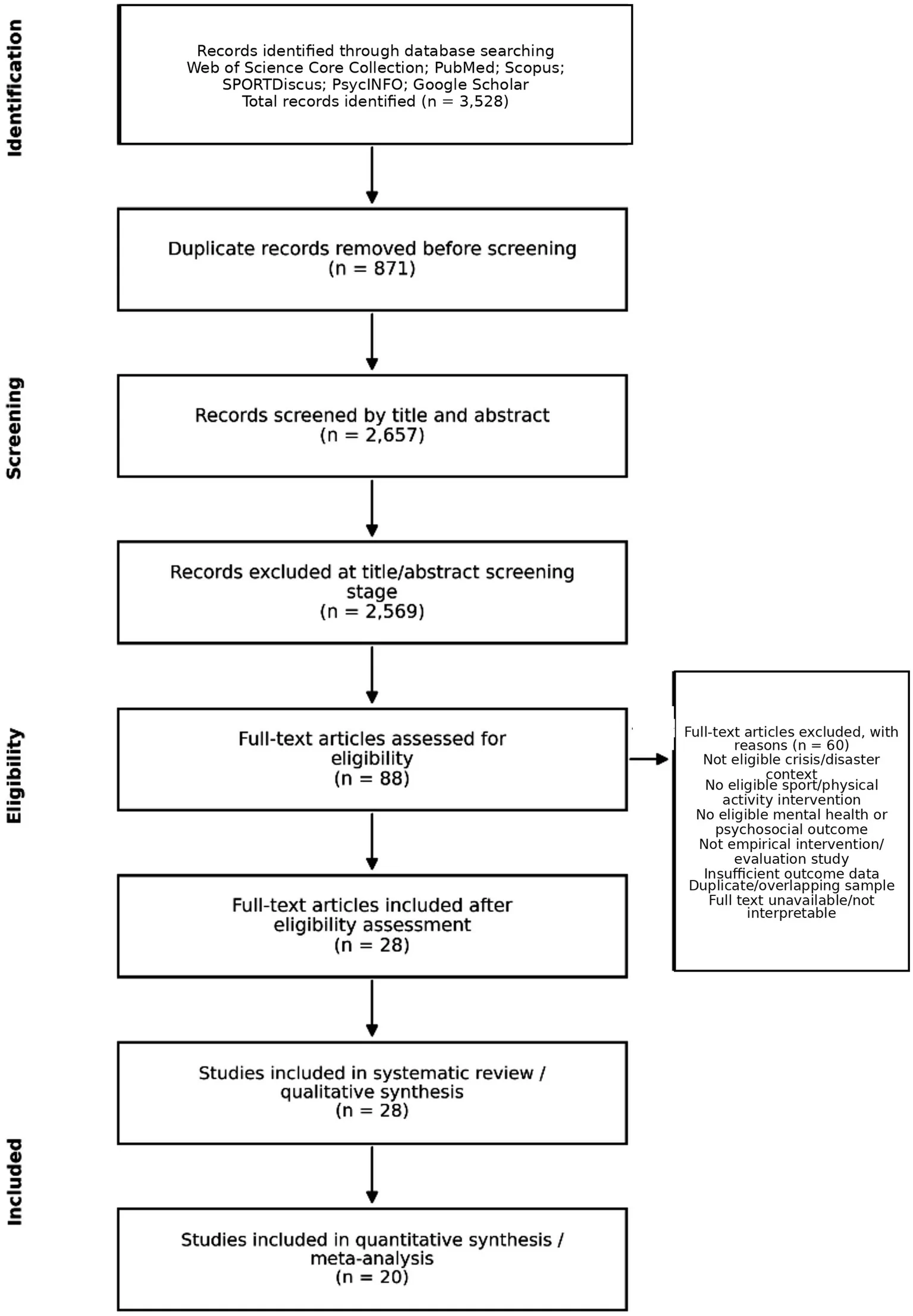
PRISMA 2020 flow diagram of study selection.

### 2.4 Rationale for separate and integrated synthesis

The quantitative and narrative components answered different but complementary questions. Meta-analysis was restricted to outcomes that were sufficiently comparable in construct and reporting—principally PTSD, depression, and anxiety—and estimated the average direction and magnitude of symptom change. Narrative synthesis addressed psychosocial constructs for which statistical pooling would have obscured substantive differences in level of analysis, measurement, and programme purpose. It also examined mechanisms, feasibility, cultural adaptation, safeguarding, and implementation conditions. The two streams were integrated after separate analysis by mapping each pooled symptom outcome against recurrent psychosocial mechanisms and contextual conditions; convergence, complementarity, and discordance were then examined in an integration matrix (Table 2).

**Table 2:** Integration of quantitative and narrative findings.

Quantitative finding	Complementary psychosocial evidence	Integrated interpretation	Boundary/uncertainty
PTSD: *g* = −0.32; *I*^2^ = 58%	Body awareness, emotional regulation, predictable routine, and safe group participation were recurrent mechanisms.	Sport-based programmes may support arousal regulation and perceived control, but the pooled symptom effect is not evidence that all programme formats are trauma treatment.	Moderate heterogeneity; mixed ages, settings, measures, and designs; controlled and uncontrolled evidence must remain separate.
Depression: *g* = −0.44; *I*^2^ = 47%	Achievement, routine, social contact, belonging, and participation were commonly reported.	Behavioural activation and social reconnection provide plausible complementary pathways for improvement.	Context and comparator varied; psychosocial mechanisms were not consistently tested as mediators.
Anxiety: *g* = −0.41; *I*^2^ = 52%	Safe social space, structured activity, coping skills, and trusted peer/adult relationships were emphasized.	Physiological regulation and environmental predictability may operate together; causation of specific mechanisms cannot be inferred.	Outcome measures and programme content differed; some outcome sets were small.
No pooled community-resilience effect	Narrative evidence described connectedness, life skills, teamwork, communication, and cohesion.	These findings extend the symptom results by showing possible social recovery functions rather than additional clinical effects.	Constructs were heterogeneous and often assessed in qualitative or non-randomized studies; confidence is therefore limited.

Negative Hedges’ g values favour the intervention for symptom outcomes. Narrative findings explain potential mechanisms and implementation conditions; they do not validate or inflate the pooled estimates.

For continuous outcomes, standardized mean differences were calculated as Hedges’ g with small-sample correction. Negative values indicate lower symptom scores in the intervention group when higher scores represented worse symptoms. The main models prioritized post-intervention between-group effects from randomized or comparative studies. Compatible change scores were used only when direction and variance could be harmonized; uncontrolled pre–post estimates were not combined with controlled effects in the primary models. Where a study reported multiple measures of the same construct or multiple intervention arms sharing a comparator, effects were coded to avoid double-counting participants. Binary outcomes were summarized as odds ratios when pooling was appropriate.

Random-effects models were selected *a priori* because true effects were expected to vary across crisis contexts, populations, intervention components, delivery settings, and measures. Between-study variance was represented by τ^2^; Cochran’s *Q* tested whether observed dispersion exceeded sampling error, and *I*^2^ described the proportion of variability attributable to heterogeneity rather than chance. *I*^2^ was interpreted as a descriptive indicator rather than a fixed threshold. Prespecified exploratory subgroup categories were intervention type, population group, and crisis context. Sensitivity analyses excluded uncontrolled designs, high-risk-of-bias studies, and overlapping samples. Publication bias procedures were used only when the number of studies was adequate; with small outcome sets, funnel plots, Egger’s test, and trim-and-fill were treated as low-power, exploratory diagnostics rather than proof that bias was absent. Meta-analysis was conducted in Comprehensive Meta-Analysis software. Effect-size estimation followed established meta-analytic procedures (62). The structured narrative component was guided by principles for transparent narrative synthesis (72). Table 2 is cited here because it provides the integration matrix used to bring together the separately synthesized quantitative and narrative evidence streams.

## 3 Results

A total of 28 independent studies or programme evaluations met the systematic-review criteria. Twenty contributed extractable data to at least one quantitative analysis, although the number contributing to each primary controlled outcome model was smaller and varied by outcome. Reports that were qualitative, implementation-focused, protocol-only, insufficiently reported for effect-size calculation, or based on an already represented sample contributed only to narrative or contextual synthesis. Accordingly, “20 studies eligible for quantitative analysis” should not be read as 20 independent studies in every outcome model.

### 3.1 Study characteristics

As a result of the search, 3,528 records were identified; after duplicates were removed, 2,657 studies were evaluated in terms of title and abstract. Eighty-eight full texts were examined, and 60 studies that did not meet the criteria were excluded. A total of 28 studies were included in the systematic review and qualitative synthesis. Of these, 20 studies provided sufficient quantitative data and were included in the meta-analysis, while the remaining studies contributed to narrative synthesis, meta- synthesis, or contextual interpretation because of qualitative design, protocol status, insufficient quantitative outcome data, or risk of double counting.

The included studies were organized into synthesis categories according to crisis context, population group, intervention type, study design, and role in the overall synthesis. Unlike a grouped presentation, each citation is presented separately in Table 3 to make clear how individual studies contributed to the qualitative synthesis, narrative synthesis, conditional quantitative synthesis, subgroup analysis, sensitivity analysis, or main meta-analysis. Randomized controlled trials and comparative quantitative studies were prioritized for the main quantitative synthesis. Uncontrolled pre–post studies were considered only in sensitivity or separate pre–post analyses. Qualitative studies, field reports, implementation reports, and protocols were not treated as equivalent to controlled outcome studies, but they were retained to clarify programme mechanisms, feasibility, cultural adaptation, implementation barriers, and contextual conditions. Secondary analyses derived from the same trial were reported separately and were not double-counted in pooled outcome models.

**Table 3:** Characteristics of included studies.

Synthesis category	Citation	Crisis/disaster context	Intervention type	Study design	Use in synthesis
Child- and youth-focused sport/play interventions after earthquakes	Kunz (47)	Bam earthquake, Iran	Sport- and play-based psychosocial intervention	Case study/programme evaluation	Narrative synthesis and mechanism interpretation
Child- and youth-focused sport/play interventions after earthquakes	Swiss Academy for Development (2005/2006) (80)	Earthquake-affected children and youth	Sport and play activities	Field report/implementation report	Narrative synthesis and contextual interpretation
Child- and youth-focused sport/play interventions after earthquakes	Joshaghani et al. (53)	Bam earthquake, Iran	Supportive art and sport intervention with group-based psychosocial content	Field trial / controlled intervention study	Conditional quantitative synthesis and PTSD-related narrative synthesis
Child- and youth-focused sport/play interventions after earthquakes	Thordardottir et al. (44)	Earthquake-affected population	Yoga/body-awareness-related intervention	Intervention study	Conditional quantitative synthesis for PTSD-related outcomes
Child- and youth-focused sport/play interventions after earthquakes	Canpolat et al. (54)	Türkiye earthquakes	Sport-, yoga-, or art/sport-supported psychosocial practices	Intervention study	Conditional quantitative synthesis and narrative synthesis
School-based physical education programmes for children at disaster risk	Nopembri and Sugiyama (55)	Merapi Volcano disaster-risk area, Indonesia	Physical education and sport as psychosocial support	Theoretical/conceptual study	Narrative synthesis and conceptual framing
School-based physical education programmes for children at disaster risk	Nopembri et al. (40)	Disaster-prone school context, Indonesia	Physical education and sport programme	Quasi-experimental / school-based intervention study	Quantitative synthesis or subgroup analysis for emotional outcomes
School-based physical education programmes for children at disaster risk	Nopembri et al. (48)	Merapi Volcano disaster-risk area, Indonesia	Physical education and sport programme targeting communication and social awareness	School-based intervention/programme development study	Narrative synthesis of psychosocial recovery outcomes
School-based physical education programmes for children at disaster risk	Nopembri et al. (49)	Merapi Volcano disaster-risk area, Indonesia	Cooperative physical education and sports lesson	School-based intervention study	Narrative synthesis of coping, problem-solving, and resilience outcomes
Exercise/sport interventions for refugees and asylum seekers	Knappe et al. (38)	Refugee camp, Greece	Eight-week exercise and sport intervention	Uncontrolled pre–post intervention study	Sensitivity analysis / separate pre–post synthesis; not included in the main controlled model
Exercise/sport interventions for refugees and asylum seekers	Knappe et al. (50)	Refugee camp, Greece	Exercise and sport implementation programme	Professional practice/implementation report	Narrative synthesis of feasibility, implementation barriers, and sustainability
Exercise/sport interventions for refugees and asylum seekers	Gerber et al. (56)	Refugee camp, Greece	Exercise and sport intervention protocol	Study protocol	Contextual information only; not included in outcome meta-analysis
Exercise/sport interventions for refugees and asylum seekers	Nordbrandt et al. (41)	Trauma-affected refugees, Denmark	Basic body awareness therapy or mixed physical activity added to treatment as usual	Pragmatic randomized controlled trial	Main quantitative synthesis for PTSD, depression, and anxiety outcomes
Exercise/sport interventions for refugees and asylum seekers	Filippou et al. (37)	Asylum-seeker camp context	Exercise and sport intervention	Pragmatic randomized controlled trial	Main quantitative synthesis for PTSD and related mental health outcomes
Exercise/sport interventions for refugees and asylum seekers	Nilsson et al. (57)	Trauma-affected refugees	Physical activity and exercise treatment	Qualitative study	Narrative synthesis of participant experiences and implementation mechanisms
Exercise/sport interventions for refugees and asylum seekers	Nilsson et al. (58)	Trauma-affected refugees	Physical activity in relation to PTSD and trauma exposure	Observational study	Contextual synthesis of physical activity, trauma exposure, and mental health
Sport-for-protection programmes for youth affected by displacement	Budde et al. (59)	Refugee and displaced youth in humanitarian settings	Sport-for-protection / movement-based psychosocial support	Protocol/programme design study	Contextual information only; not included in outcome meta-analysis
Sport-for-protection programmes for youth affected by displacement	Ahmed (45)	Rohingya adolescents in a forced-migration and refugee context	Sports and physical activity-based psychosocial support	Field evaluation/qualitative report	Narrative synthesis of cultural adaptability, safe social space, and psychosocial support
Sport-for-protection programmes for youth affected by displacement	Latimer et al. (39)	Displaced and host-community youth, Uganda	Game Connect sport-for-protection programme	Randomized controlled trial	Main quantitative synthesis for depression and anxiety outcomes
Sport-for-protection programmes for youth affected by displacement	Latimer et al. (52)	Displaced and host-community youth, Uganda	Game Connect sport-for-protection programme	Secondary analysis of randomized trial data	Narrative synthesis for suicidal ideation; not double-counted in the main pooled model
Sport-for-protection programmes for youth affected by displacement	Jordans et al. (46)	Refugee children, Northern Uganda	Movement-based mental health promotion intervention/TeamUp-related programme	Follow-up and mediation study	Narrative synthesis and mechanism interpretation
Sport-for-protection programmes for youth affected by displacement	Bleile et al. (51)	Refugee children, Northern Uganda	Movement-based mental health promotion intervention	Evaluation study	Narrative synthesis of child-focused movement-based psychosocial support
Physical activity and online exercise interventions in the pandemic context	Beauchamp et al. (36)	COVID-19 pandemic, Canada	Online-delivered group and personal exercise programmes	Randomized controlled trial	Subgroup synthesis for depression, anxiety, and psychological well-being
Physical activity and online exercise interventions in the pandemic context	Puterman et al. (43)	COVID-19 pandemic, Canada	App-based at-home exercise programmes	Multigroup pragmatic randomized controlled trial	Subgroup synthesis for depressive symptoms and psychological well-being
Physical activity and online exercise interventions in the pandemic context	Philippot et al. (42)	COVID-19 lockdown, Belgium	Online high-intensity interval training	Randomized controlled feasibility trial	Subgroup synthesis for psychological symptoms
Physical activity and online exercise interventions in the pandemic context	Barğı (35)	COVID-19 distance-learning context, Türkiye	Physical activity counselling	Randomized controlled trial	Subgroup synthesis for depression, anxiety, and psychological well-being
Additional contextual and community-based evidence	Qiu et al. (78)	Student population in a crisis-relevant psychosocial context	Physical activity level	Observational / correlational study	Narrative synthesis of physical activity–resilience relationship
Additional contextual and community-based evidence	Rehal (77)	Post-typhoon Leyte, Philippines	Football-based psychosocial intervention	Field report / programme evaluation	Narrative synthesis of community-based sport intervention

### 3.2 Mental health outcomes

Mental health outcomes were limited to individual symptom or risk indicators: PTSD, depression, anxiety, and suicidal ideation. PTSD, depression, and anxiety were considered for outcome-specific pooling; suicidal ideation was represented by a secondary analysis from one programme family and was therefore synthesized narratively. Psychosocial outcomes—including routine, emotional regulation, communication, belonging, social connectedness, life skills, and community cohesion—were not relabelled as mental health effects. This distinction prevents improvement in a social or developmental construct from being interpreted as equivalent to clinical symptom reduction.

The effects of sport- and physical activity-based interventions on mental health were evaluated through outcomes related to PTSD symptoms, depression, anxiety, suicidal ideation, and psychosocial recovery. The findings show that structured exercise, yoga/body-awareness practices, school-based physical education, online or home-based exercise, physical activity counselling, and sport-for-protection programmes generally produced results in favour of the intervention in terms of mental health symptoms, although the magnitude and consistency of effects differed across study designs and crisis contexts (35–43). Yoga and body-awareness-oriented approaches were particularly relevant for PTSD and stress-related symptoms, whereas structured exercise, online exercise, and sport-for-protection programmes contributed mainly to depression and anxiety outcomes (36, 39, 41, 42, 44). However, due to heterogeneity in study design, sample characteristics, crisis context, intervention content, comparator structure, and measurement tools, the results need to be interpreted separately according to outcome domains. While quantitative synthesis was possible for PTSD symptoms, depression, and anxiety, variables such as positive mood, social connectedness, communication, problem-solving, and psychosocial recovery were evaluated mostly within the scope of narrative synthesis and meta-synthesis (45–49) (see Table 4).

**Table 4:** Effects on mental health outcomes by individual citation.

Mental health outcome	Citation	Intervention type	General finding
PTSD symptoms	Joshaghani et al. (53)	Supportive art and sport intervention with group-based psychosocial content	A reduction in earthquake-related post-traumatic stress symptoms was reported among children exposed to the Bam earthquake.
PTSD symptoms	Thordardottir et al. (44)	Yoga/body-awareness-related intervention	PTSD and stress-related symptoms tended to decrease following the intervention.
PTSD symptoms	Knappe et al. (38)	Eight-week exercise and sport intervention	Improvements were reported in PTSD symptoms and broader mental health indicators among refugees living in a Greek refugee camp.
PTSD symptoms	Nordbrandt et al. (41)	Basic body awareness therapy or mixed physical activity added to treatment as usual	The intervention did not show clear superiority over treatment as usual for PTSD symptoms.
PTSD symptoms	Filippou et al. (37)	Exercise and sport intervention in an asylum-seeker camp	The intervention was evaluated for PTSD and related mental health outcomes in a camp setting.
Depression	Nopembri et al. (40)	School-based physical education and sport programme	Improvement in emotional and psychosocial indicators was reported in the intervention context.
Depression	Knappe et al. (38)	Eight-week exercise and sport intervention	Depressive symptoms improved after participation in the exercise and sport programme.
Depression	Nordbrandt et al. (41)	Basic body awareness therapy or mixed physical activity added to treatment as usual	Depression-related outcomes did not show a consistently strong additional benefit over treatment as usual.
Depression	Puterman et al. (43)	App-based or home-based exercise programme during the COVID-19 pandemic	Depressive symptoms and psychological well-being outcomes were examined in the context of pandemic restrictions.
Depression	Beauchamp et al. (36)	Online-delivered group and personal exercise programme	The intervention showed favorable effects on mental health and psychological well-being among low-active older adults during the COVID-19 pandemic.
Depression	Philippot et al. (42)	Online high-intensity interval training	Depression-related psychological symptoms were assessed following an online exercise intervention during lockdown.
Depression	Barğı (35)	Physical activity counselling	Physical activity counselling was associated with favorable mental health outcomes among university students in a distance-learning context.
Depression	Latimer et al. (39)	Game Connect sport-for-protection programme	The intervention reduced depressive symptoms among displaced and host-community youth.
Anxiety	Nopembri et al. (40)	Psychosocial physical education and sport programme	Anxiety-related emotional outcomes tended to improve in the intervention group.
Anxiety	Knappe et al. (38)	Exercise and sport intervention	Anxiety symptoms improved following the exercise and sport programme.
Anxiety	Latimer et al. (39)	Game Connect sport-for-protection programme	Anxiety symptoms decreased among youth affected by displacement.
Anxiety	Barğı (35)	Physical activity counselling	Anxiety-related outcomes improved in the physical activity counselling context.
Suicidal ideation	Latimer et al. (52)	Game Connect sport-for-protection programme	A reduction in suicidal ideation was reported among youth affected by displacement.
Positive mood and psychosocial recovery	Kunz (47)	Sport- and play-based psychosocial intervention	Emotional expression, social reconnection, teamwork, and safe group experience were supported among children and youth after the Bam earthquake.
Positive mood and psychosocial recovery	Nopembri et al. (48)	Physical education and sport programme targeting communication and social awareness	Communication, social awareness, and group-based psychosocial functioning were supported.
Positive mood and psychosocial recovery	Nopembri et al. (49)	Cooperative physical education and sport lesson	Coping with stress, problem-solving, cooperation, and social interaction were supported.
Positive mood and psychosocial recovery	Ahmed (45)	Sports and physical activity-based psychosocial support	Safe social space, cultural adaptability, and psychosocial support were emphasized among Rohingya adolescents.
Positive mood and psychosocial recovery	Jordans et al. (46)/TeamUp-related studies	Movement-based mental health promotion intervention	Group-based movement activities supported psychosocial functioning among refugee children.

### 3.3 Psychosocial recovery and community resilience

Table 5 summarizes non-clinical psychosocial and community-level evidence by citation. These findings were not pooled because the studies differed in construct, unit of analysis, design, and measurement: for example, communication skills, safe social space, life skills, and community cohesion are related but not statistically interchangeable outcomes. The narrative synthesis therefore examined recurring mechanisms and implementation conditions rather than calculating a single “psychosocial recovery” effect. Across earthquake, displacement, refugee-camp, and disaster-risk settings, recurrent themes included restoration of routine, emotional expression, peer connection, belonging, teamwork, coping, and participation. Confidence in these themes is lower than confidence in a replicated pooled effect because much of the evidence was qualitative, uncontrolled, or programme-specific.

**Table 5:** Psychosocial recovery and community resilience outcomes.

Analysis dimension	Citation/study source	Population and context	Intervention/variable	Meta-synthesis code
Psychological resilience	Qiu et al. (78)	Young students in a crisis-relevant psychosocial context	Physical activity level	Physical activity–resilience relationship
Social connectedness and community cohesion	Kunz (80)	Children and youth affected by the Bam earthquake	Sport- and play-based psychosocial intervention	Emotional release and social reconnection
Social connectedness and community cohesion	Kunz (47)	Children and youth affected by the Bam earthquake	Sport- and play-based psychosocial intervention	Social connectedness and community cohesion
Teamwork and fair play	Kunz (80)	Children after the Bam earthquake	Play, sport, and group-based activities	Collective resilience skills
Teamwork and fair play	Kunz (47)	Children and youth after the Bam earthquake	Play, sport, and group-based activities	Cooperation, trust, and shared group experience
Life skills	Latimer et al. (39)	Youth affected by displacement and host-community youth in Uganda	Game Connect sport-for-protection programme	Life skills-based psychosocial recovery
Community resilience	Latimer et al. (39)	Refugee, displaced, and host-community youth in Uganda	Sport-based mental health and psychosocial support programme	Sport-for-protection and community capacity
Community resilience	Olympic Refuge Foundation and Right To Play programme sources	Refugee and host-community youth in Uganda	Game Connect sport-for-protection model	Community-based sport-for-protection capacity
Communication and social awareness	Nopembri et al. (48)	Primary school students at risk of the Merapi Volcano disaster	Physical education and sport programme	Social skills development
Coping with stress and problem-solving	Nopembri et al. (49)	Children in the Merapi disaster-risk region	Cooperative physical education and sport lesson	Coping capacity and problem-solving
Safe social space	Ahmed (45)	Rohingya adolescents in a forced-migration and refugee context	Sport- and physical activity-based psychosocial support	Cultural adaptability and safe group space
Movement-based mental health promotion	Jordans et al. (46)	Refugee children in Northern Uganda	Movement-based group intervention / TeamUp-related programme	Group-based psychosocial support
Movement-based mental health promotion	Bleile et al. (51)	Refugee children in Northern Uganda	Movement-based mental health promotion intervention	Child-focused movement-based psychosocial support
Implementation sustainability	Knappe et al. (50)	Refugee camp in Greece	Implementation of an exercise and sport programme	Implementation context and sustainability
Social participation and humanitarian crisis context	Rehal (77)	Post-typhoon Leyte, Philippines	Football-based psychosocial intervention	Community-based sport intervention

Table 5 shows that the effect of sport- and physical activity-based interventions on post-disaster psychosocial recovery is not limited solely to the reduction of individual mental health symptoms. The findings reveal that physical activity may have a supportive function in multidimensional domains such as psychological resilience, social connectedness, emotional release, communication, problem-solving, teamwork, fair play, safe group experience, life skills, and community participation (39, 45–50, 78). In particular, evidence from the Bam earthquake programme, the Game Connect sport-for-protection programme, the Merapi disaster-risk school-based physical education studies, and TeamUp-related movement-based interventions suggests that sport may contribute to the re-establishment of relationships within the community after disaster or displacement by reducing experiences of loneliness, insecurity, and social disconnection and by strengthening belonging, trust, peer support, and shared routine (39, 46–49, 51). However, these effects depend not on sport being inherently therapeutic, but rather on the intervention being designed in a safe, culturally adapted, age-appropriate manner and integrated with psychosocial support principles. Therefore, the studies in question indicate that sport-based interventions can be considered complementary psychosocial support tools that may strengthen post-disaster community resilience when embedded within broader MHPSS systems.

Table 6 is an outcome-contribution table, not a table of study-specific effects. The pooled estimate is repeated within an outcome solely to show the model to which each citation contributed; it must not be interpreted as the individual effect of every listed study. A study could contribute to more than one outcome when independent, extractable data were reported. Secondary reports from the same trial were not double-counted, and uncontrolled pre–post studies were excluded from the primary controlled estimates and retained only in separate sensitivity or descriptive analyses.

**Table 6:** Summary of meta-analysis results.

Outcome	Citation included in quantitative synthesis	Population and sample size	Intervention/comparator	Relevant measurement tool	Outcome-level pooled estimate (repeated to identify model)
PTSD symptoms	Joshaghani et al. (53)	Children exposed to the Bam earthquake; approximately 200 randomized participants, with exclusions reported in the original study	Group-based behavioural, art-supported, and sport-supported psychosocial intervention arms compared with control conditions	PTSD diagnosis/symptom assessment based on child psychiatric interview procedures reported in the original study	Hedges’ *g* = −0.32, 95% CI [−0.50, −0.14], *p* < 0.01; *I*^2^ = 58%, Tau^2^ = 0.04
PTSD symptoms	Thordardottir et al. (44)	Earthquake-exposed adults in South Iceland; *n* = 66, yoga group *n* = 31 and waitlist control *n* = 35	Yoga intervention compared with waitlist control	Validated measures of posttraumatic symptoms, depression, anxiety, stress, and health-related quality of life as reported in the original study	Hedges’ *g* = −0.32, 95% CI [−0.50, −0.14], *p* < 0.01; *I*^2^ = 58%, Tau^2^ = 0.04
PTSD symptoms	Nordbrandt et al. (41)	Adult trauma-affected refugees with PTSD in Denmark; *n* = 338 randomized and *n* = 318 included in intention-to-treat analyses	Basic body awareness therapy or mixed physical activity added to treatment as usual, compared with treatment as usual alone	Harvard Trauma Questionnaire for PTSD; Hamilton Depression Rating Scale and Hamilton Anxiety Rating Scale for secondary mental health outcomes	Hedges’ *g* = −0.32, 95% CI [−0.50, −0.14], *p* < 0.01; *I*^2^ = 58%, Tau^2^ = 0.04
PTSD symptoms	Filippou et al. (37)	Asylum seekers living in a camp context; *n* = 142 randomized and *n* = 98 completed the intervention assessment	Ten-week exercise and sport intervention compared with control/usual camp activities	Impact of Event Scale-Revised for PTSD symptoms; additional measures included depression, anxiety, stress, and well-being	Hedges’ *g* = −0.32, 95% CI [−0.50, −0.14], *p* < 0.01; *I*^2^ = 58%, Tau^2^ = 0.04
Depression	Nopembri et al. (40)	Fourth- to sixth-grade students in disaster-prone schools near Merapi Volcano, Indonesia; 15 physical education teachers and 810 students	School-based physical education and sports programme with psychosocial practices compared with existing physical education programmes	Depression, Anxiety and Stress Scales-42 depression subscale	Hedges’ *g* = −0.44, 95% CI [−0.60, −0.28], *p* < 0.001; *I*^2^ = 47%, Tau^2^ = 0.03
Depression	Nordbrandt et al. (41)	Adult trauma-affected refugees with PTSD in Denmark; *n* = 338 randomized and *n* = 318 intention-to-treat	Basic body awareness therapy or mixed physical activity plus treatment as usual compared with treatment as usual alone	Hamilton Depression Rating Scale	Hedges’ *g* = −0.44, 95% CI [−0.60, −0.28], *p* < 0.001; *I*^2^ = 47%, Tau^2^ = 0.03
Depression	Puterman et al. (43)	Low-active adults during the COVID-19 pandemic; *n* = 334 randomized	Six-week home-based HIIT, yoga, combined HIIT plus yoga, or waitlist control	Center for Epidemiologic Studies Depression Scale, 10-item version	Hedges’ *g* = −0.44, 95% CI [−0.60, −0.28], *p* < 0.001; *I*^2^ = 47%, Tau^2^ = 0.03
Depression	Beauchamp et al. (36)	Low-active older adults during the COVID-19 pandemic; *n* = 241 randomized	Online-delivered group exercise or personal exercise programme compared with control	Center for Epidemiologic Studies Depression Scale, 10-item version	Hedges’ *g* = −0.44, 95% CI [−0.60, −0.28], *p* < 0.001; *I*^2^ = 47%, Tau^2^ = 0.03
Depression	Philippot et al. (42)	Young adults/students during COVID-19 lockdown; *n* = 30 randomized, with *n* = 28 completing post-intervention assessment	Four-week online high-intensity interval training compared with control	Depression, Anxiety and Stress Scales-21 depression subscale	Hedges’ *g* = −0.44, 95% CI [−0.60, −0.28], *p* < 0.001; *I*^2^ = 47%, Tau^2^ = 0.03
Depression	Barğı (35)	University students educated by distance learning during the COVID-19 pandemic; physical activity counselling group *n* = 15 and control group *n* = 16	Four-week physical activity counselling based on progressive increases in step counts compared with routine activity control	Beck Depression Inventory	Hedges’ *g* = −0.44, 95% CI [−0.60, −0.28], *p* < 0.001; *I*^2^ = 47%, Tau^2^ = 0.03
Depression	Latimer et al. (39)	Displaced and host-community youth in Uganda; *n* = 834 randomized	Game Connect sport-for-protection programme compared with waitlist/control condition	Patient Health Questionnaire-9, adolescent version	Hedges’ *g* = −0.44, 95% CI [−0.60, −0.28], *p* < 0.001; *I*^2^ = 47%, Tau^2^ = 0.03
Anxiety	Nopembri et al. (40)	Fourth- to sixth-grade students in disaster-prone schools near Merapi Volcano, Indonesia; 15 teachers and 810 students	School-based physical education and sports programme with psychosocial practices compared with existing physical education programmes	Depression, Anxiety and Stress Scales-42 anxiety subscale	Hedges’ *g* = −0.41, 95% CI [−0.58, −0.24], *p* < 0.001; *I*^2^ = 52%, Tau^2^ = 0.03
Anxiety	Nordbrandt et al. (41)	Adult trauma-affected refugees with PTSD in Denmark; *n* = 338 randomized and *n* = 318 intention-to-treat	Basic body awareness therapy or mixed physical activity plus treatment as usual compared with treatment as usual alone	Hamilton Anxiety Rating Scale	Hedges’ *g* = −0.41, 95% CI [−0.58, −0.24], *p* < 0.001; *I*^2^ = 52%, Tau^2^ = 0.03
Anxiety	Filippou et al. (37)	Asylum seekers in a camp context; *n* = 142 randomized and *n* = 98 completed assessment	Ten-week exercise and sport intervention compared with control/usual camp activities	Generalized Anxiety Disorder-7 or anxiety measure reported in the original article	Hedges’ *g* = −0.41, 95% CI [−0.58, −0.24], *p* < 0.001; *I*^2^ = 52%, Tau^2^ = 0.03
Anxiety	Philippot et al. (42)	Young adults/students during COVID-19 lockdown; *n* = 30 randomized, with *n* = 28 completing post-intervention assessment	Four-week online high-intensity interval training compared with control	Depression, Anxiety and Stress Scales-21 anxiety subscale; Generalized Anxiety Disorder-7 used for eligibility/screening	Hedges’ *g* = −0.41, 95% CI [−0.58, −0.24], *p* < 0.001; *I*^2^ = 52%, Tau^2^ = 0.03
Anxiety	Barğı (35)	University students educated by distance learning during the COVID-19 pandemic; physical activity counselling group *n* = 15 and control group *n* = 16	Four-week physical activity counselling based on progressive increases in step counts compared with routine activity control	Beck Anxiety Inventory	Hedges’ *g* = −0.41, 95% CI [−0.58, −0.24], *p* < 0.001; *I*^2^ = 52%, Tau^2^ = 0.03
Anxiety	Latimer et al. (39)	Displaced and host-community youth in Uganda; *n* = 834 randomized	Game Connect sport-for-protection programme compared with waitlist/control condition	Generalized Anxiety Disorder-7	Hedges’ *g* = −0.41, 95% CI [−0.58, −0.24], *p* < 0.001; *I*^2^ = 52%, Tau^2^ = 0.03

Eligibility for quantitative synthesis was determined at the outcome level. Randomized and comparative quantitative studies with an independent comparator and extractable data were prioritized for the primary models. Uncontrolled pre–post studies, protocols, qualitative studies, field reports, implementation evaluations, and secondary reports from already represented samples were not pooled in the main controlled estimates. This design-based separation was necessary because combining within-person change with between-group effects would change the estimand and could exaggerate apparent intervention benefit.

### 3.4 Risk of bias and study quality

Risk of bias varied materially by design and affects the certainty—not merely the description—of the review conclusions. Randomized trials provided the strongest basis for causal inference, but common concerns included unclear allocation concealment, the impossibility of participant and provider blinding in behavioural programmes, attrition, adherence, and selective or incomplete outcome reporting. Lack of participant blinding was not treated automatically as fatal; its importance depended on whether outcomes were self-reported, whether outcome assessors could be blinded, and whether deviations from the intended intervention were balanced across groups. Non-randomized comparative studies were more vulnerable to baseline imbalance, confounding, selection into participation, and differential co-intervention. Uncontrolled pre–post studies could describe change but could not separate intervention effects from spontaneous recovery, regression to the mean, historical events, or concurrent support. Qualitative and implementation studies were not entered into the quantitative risk-of-bias model and were used only for mechanisms, acceptability, feasibility, and context (see Table 7).

**Table 7:** Eligibility logic for meta-analysis.

Eligibility group	Relevant citations	Use in the meta-analysis	Rationale
Studies suitable for the main controlled quantitative model	Nordbrandt et al. (41); Filippou et al. (37); Latimer et al. (39); Puterman et al. (43); Beauchamp et al. (36); Philippot et al. (42); Barğı (35); Nopembri et al. (40)	Main meta-analysis or outcome-specific subgroup analysis	These studies reported quantitative mental health outcomes and had a randomized, comparative, quasi-experimental, or controlled structure that allowed extraction of intervention and comparison data.
Studies conditionally eligible for quantitative synthesis	Joshaghani et al. (53); Thordardottir et al. (44); Canpolat et al. (54); Jordans et al. (46)/TeamUp-related evaluation studies	Included only when outcome data, comparator structure, and independence of sample were compatible with the outcome model; otherwise retained for narrative synthesis	These studies provided relevant intervention evidence but required cautious coding because of mixed intervention content, limited sample size, incomplete reporting, context-specific outcomes, or potential overlap with programme-family data.
Studies suitable only for sensitivity or separate pre–post analysis	Knappe et al. (38)	Sensitivity analysis or separate pre–post change synthesis; not included in the main controlled model	The study reported relevant PTSD, depression, and anxiety outcomes, but the uncontrolled pre–post design did not allow causal comparison with a control group.
Studies suitable for qualitative synthesis, narrative synthesis, or meta-synthesis	Swiss Academy for Development (2005/2006) from Kunz (80); Ahmed (45); Knappe et al. (50); Nilsson et al. (57); Nopembri and Sugiyama (55); Nopembri et al. (48); Nopembri et al. (49); Rehal (77); Qiu et al. (78); Bleile et al. (51)	Narrative synthesis, meta-synthesis, contextual interpretation, mechanism interpretation, or implementation synthesis	These studies, reports, or programme evaluations contributed important evidence on feasibility, acceptability, social connectedness, community resilience, life skills, cultural adaptation, implementation barriers, and psychosocial mechanisms, but did not provide comparable quantitative outcome data suitable for the main meta-analysis.
Studies or reports not included in the outcome meta-analysis	Budde et al. (59); Gerber et al. (56); Latimer et al. (52); Nilsson et al. (58); secondary analyses derived from samples already represented in the main model	Excluded from pooled outcome models; used only for protocol/contextual information or discussed separately when relevant	Protocols did not contain post-intervention outcome data. Secondary analyses were not double-counted when they used the same sample as a primary trial already included in the main model. Observational or secondary reports were retained only for contextual interpretation when they could not provide independent intervention-control effect sizes.

These limitations temper the pooled estimates in three ways. First, favorable average effects should be interpreted as signals of benefit rather than precise predictions for a new setting. Second, moderate statistical heterogeneity is compounded by clinical and methodological diversity, so the mean effect may not represent any single intervention format or population. Third, evidence for suicidal ideation, community resilience, social connectedness, and life skills is less certain because it comes from fewer, often programme-specific or non-randomized sources. Sensitivity analyses excluding uncontrolled and high-risk studies were therefore used to assess robustness, and narrative claims were calibrated to design strength. Table 8 links the principal quality concerns to their likely influence on inference.

**Table 8:** Risk-of-bias domains and implications for interpretation.

Evidence type	Principal concerns	Likely influence	Use in conclusions
Randomized trials	Allocation concealment, attrition, adherence, self-reported outcomes, limited assessor blinding	May over- or underestimate effects; attrition and deviations may compromise comparability	Primary basis for causal interpretation, with cautious wording
Non-randomized comparative studies	Confounding, baseline imbalance, selection, co-interventions	Favorable effects may partly reflect pre-existing group differences	Supportive evidence; not treated as equivalent to low-risk randomized evidence
Uncontrolled pre–post studies	No concurrent counterfactual, regression to the mean, natural recovery, history	Change cannot be attributed confidently to the intervention	Separate sensitivity or descriptive synthesis only
Qualitative/implementation evidence	Purposive samples, researcher influence, incomplete reflexivity or fidelity reporting	Transferability and credibility of mechanisms may be limited	Used for context, feasibility, acceptability, and mechanisms—not effect magnitude

Figure 3 displays the pooled PTSD estimate and its 95% confidence interval. The estimate favors the intervention, but *I*^2^ = 58% indicates meaningful between-study variability.

**Figure 3:**
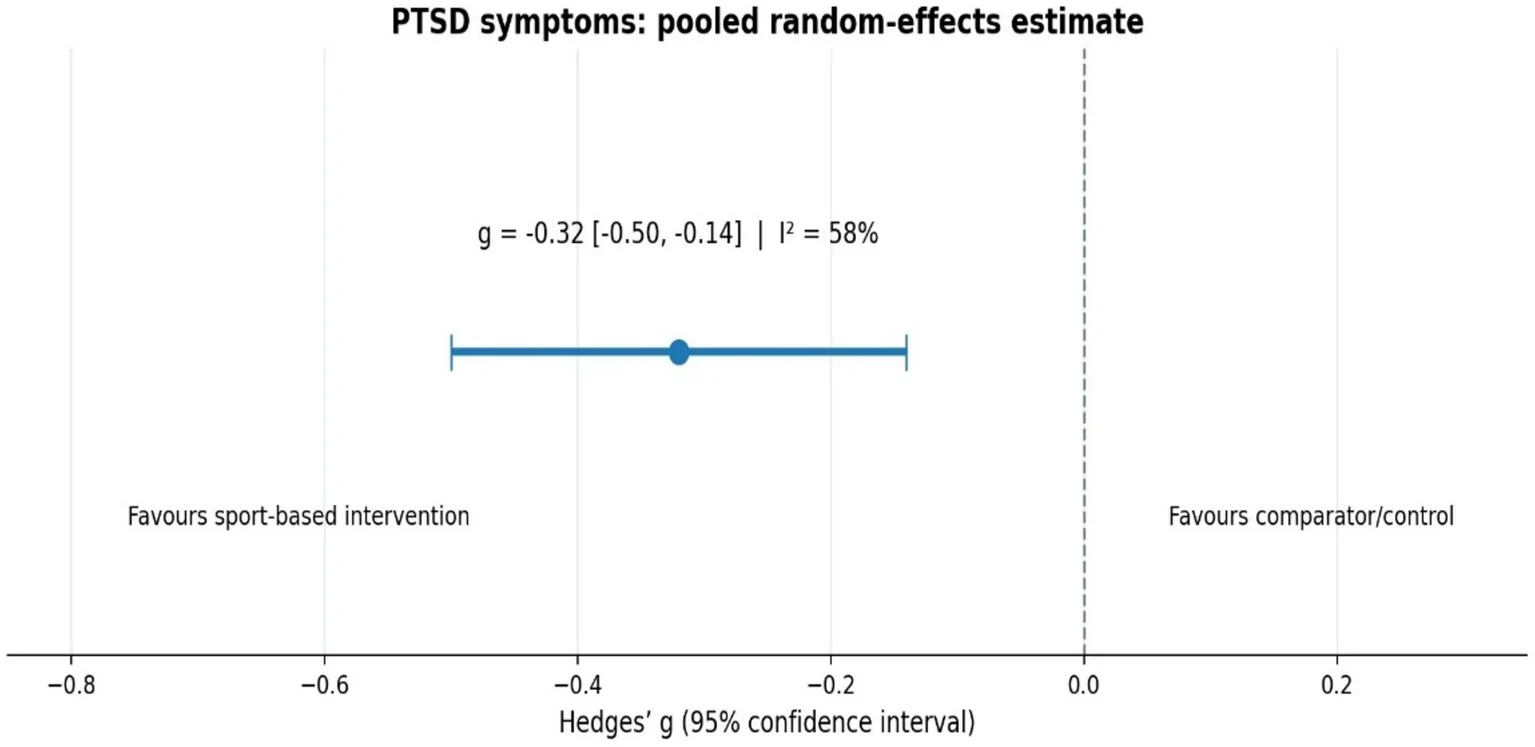
Pooled random-effects estimate for PTSD symptoms.

Figure 4 displays the pooled depression estimate and its 95% confidence interval. The estimate favors the intervention; its interpretation remains conditional on study quality, comparator, population, and programme content.

**Figure 4:**
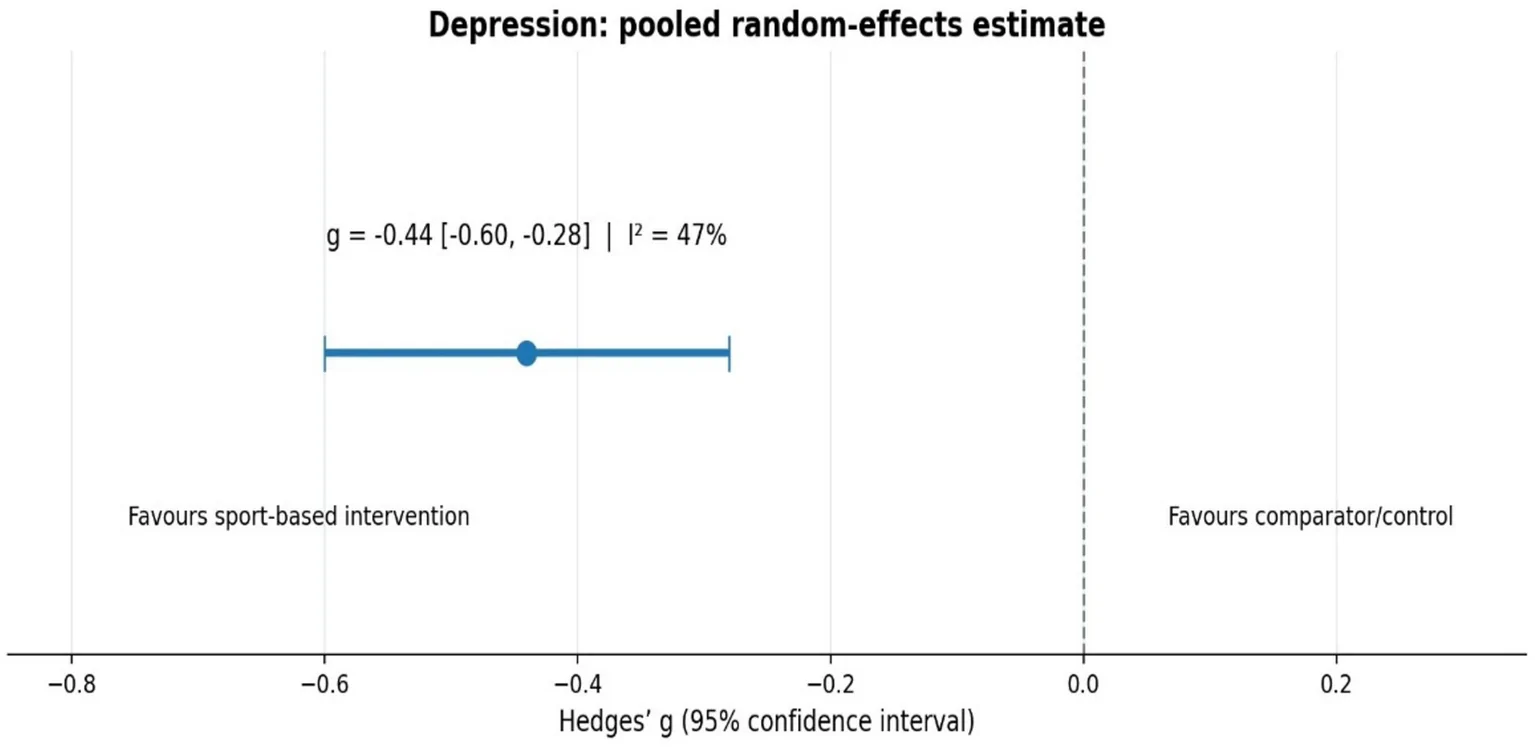
Pooled random-effects estimate for depression.

Figure 5 displays the pooled anxiety estimate and its 95% confidence interval. The favorable average effect should not be interpreted as a uniform response across settings.

**Figure 5:**
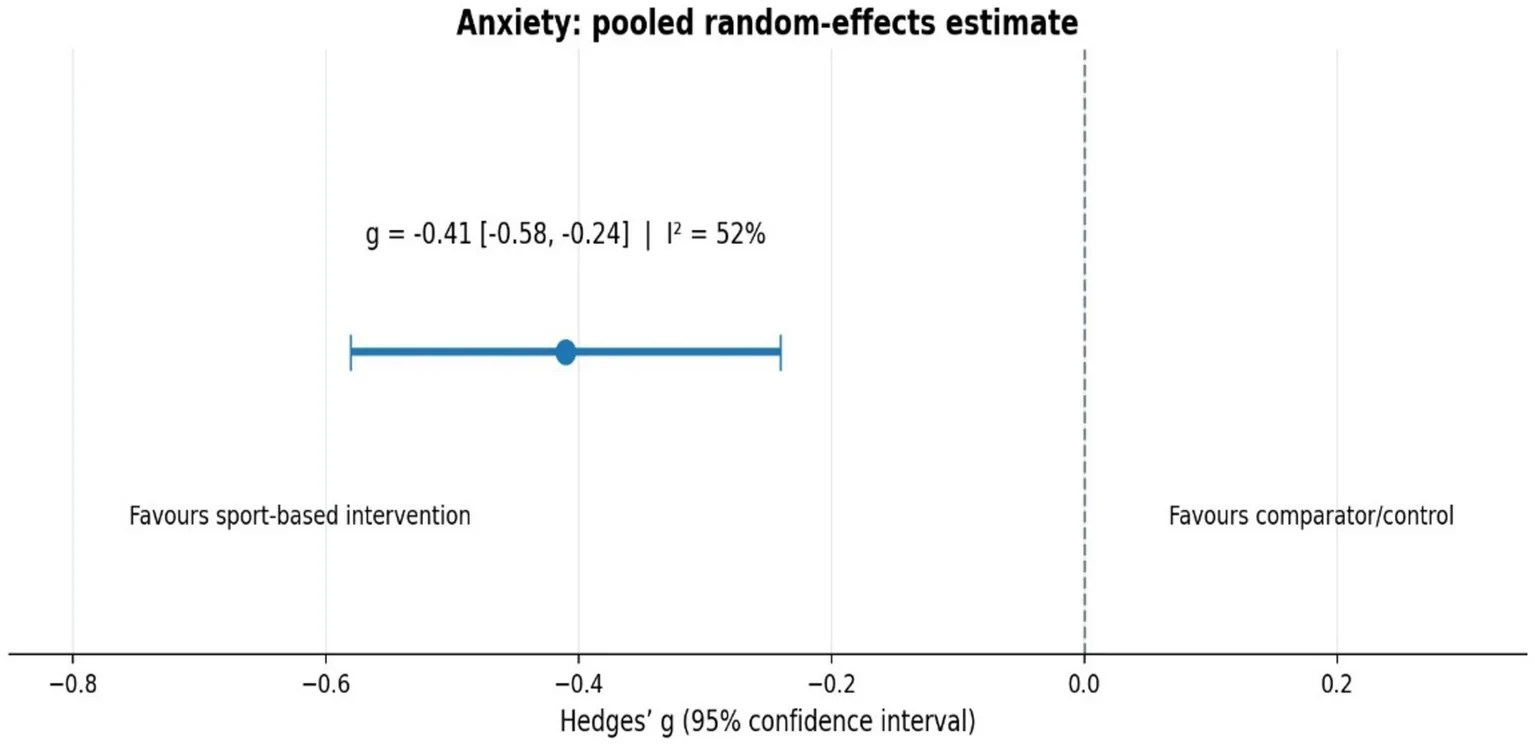
Pooled random-effects estimate for anxiety.

## 4 Discussion

### 4.1 Integrated interpretation within the MHPSS framework

The two evidence streams converge on a qualified conclusion. The meta-analysis indicates favorable average changes in PTSD, depression, and anxiety symptoms, while the narrative synthesis identifies routine, emotional regulation, social connection, belonging, life skills, and safe participation as plausible psychosocial functions. The narrative evidence therefore adds explanatory and contextual depth; it does not constitute an independent confirmation of the pooled symptom effects. Nor does convergence establish mediation, because few included studies formally tested whether changes in belonging, routine, or regulation produced symptom improvement. Within the MHPSS framework, the evidence most strongly supports sport-based programmes as community and family supports or focused, non-specialized supports that should connect—through referral pathways—to specialized care when clinical risk or severe impairment is identified (7, 8).

### 4.2 Interpretation of effect sizes and heterogeneity

The pooled effects were small-to-moderate for PTSD (*g* = −0.32), depression (*g* = −0.44), and anxiety (*g* = −0.41). These standardized effects describe average differences across dissimilar measures; they do not translate directly into diagnosis, remission, or clinically important change for an individual participant. Their public-health relevance may nevertheless be meaningful when low-threshold programmes reach populations with limited access to formal services. That potential must be balanced against *I*^2^ values of 47–58%, which indicate that the magnitude of benefit varied across settings and was not explained by sampling error alone. Differences in age, trauma exposure, displacement status, programme content and dose, comparator, adherence, and outcome measurement are plausible sources, but the available evidence does not permit confident attribution of heterogeneity to any single factor.

Study-level findings illustrate why the pooled estimates require caution. The uncontrolled refugee-camp study by Knappe et al. (38) reported improvements across several outcomes, but without a concurrent comparator the changes cannot be attributed securely to the intervention. In contrast, Nordbrandt et al. (41) found no clear additional PTSD benefit when body-awareness therapy or mixed physical activity was added to treatment as usual. More recent pragmatic trials in asylum-seeker and displacement settings show that rigorous evaluation is feasible, yet effects remain dependent on attendance, programme fidelity, comparator content, and camp or community conditions (37, 39). The pooled values should therefore be read as a promising average signal, not as evidence that every sport-based programme will produce a similar effect.

### 4.3 Complementarity of symptom and psychosocial findings

For depression and anxiety, the narrative themes of routine, achievement, social contact, and safe group participation are consistent with behavioural and social pathways through which structured activity could help. The Game Connect trial, for example, combined sport with protection and life-skills components and reported reductions in depression and anxiety among displaced and host-community youth (39). Because multicomponent interventions do not isolate the active ingredient, these results cannot be attributed to physical exertion alone. The same caution applies to PTSD: body awareness, predictable structure, and perceived control are plausible mechanisms, but they remain hypotheses unless tested in mediation or component studies.

Psychosocial findings extend the review beyond symptom reduction without being treated as clinical outcomes. Across the Bam, Merapi, Rohingya, Uganda, and Greek camp contexts, reports described emotional expression, peer connection, communication, cooperation, belonging, coping, and safe social space (45–50). These themes help explain how programmes may contribute to recovery at interpersonal and community levels. However, construct heterogeneity, qualitative designs, small or purposive samples, and limited fidelity reporting reduce certainty and transferability. Evidence concerning suicidal ideation is particularly preliminary because it derives from a secondary analysis within one programme family; it cannot support sport as a substitute for suicide-risk assessment, crisis response, or clinical care (52).

### 4.4 Conditions for safe and effective implementation

The findings do not support an assumption that sport is inherently therapeutic. Benefits are most plausible when programmes are voluntary, trauma-sensitive, developmentally appropriate, culturally adapted, accessible across gender and disability, and delivered by trained facilitators within a predictable and psychologically safe environment. Safeguarding procedures should address bullying, harassment, sexual exploitation and abuse, discriminatory team selection, injury, excessive competition, and disclosure of protection concerns. Programmes should include informed participation, confidential reporting routes, facilitator supervision, attendance and adverse-event monitoring, and clear referral pathways for severe symptoms, suicidality, child-protection concerns, or functional deterioration. Community consultation and co-design are needed to adapt activity type, clothing, mixed-gender participation, timing, language, and family involvement without diluting core safety and support functions.

Operationally, sport-based interventions should be positioned within a mapped local MHPSS system rather than delivered as isolated recreation. Schools, temporary accommodation areas, community centres, youth spaces, and rehabilitation services may be suitable platforms when basic safety and access are secured. A minimum implementation package should specify the target population and tier of support; intervention components, frequency, and dose; facilitator competencies and supervision; safeguarding and referral protocols; cultural adaptations; fidelity indicators; and mental health, psychosocial, participation, and adverse-event outcomes. Partnerships among mental health professionals, protection actors, educators, coaches, disability organizations, local authorities, and community leaders can help ensure that programmes extend access while remaining connected to focused and specialized services.

### 4.5 Limitations and research priorities

Broader literature retained in the reference list was used to delimit the scope and interpret the findings rather than to enlarge the pooled evidence base. General evidence on physical activity and mental health, trauma recovery, and child resilience informed the conceptual interpretation (64, 68, 70). Evidence from sport-for-development literature also informed the conceptual framing (66, 73, 74). Post-earthquake documentation and crisis-focused sport research helped situate the review within disaster and community settings (61, 65, 67). Evidence from severe-mental-disorder programmes, refugee trials and secondary analyses, and earthquake-focused therapeutic play was used only for contextual comparison and emerging research directions (63, 69, 71, 76). By contrast, occupational musculoskeletal prevention and family-based child-protection literature fall outside the review’s eligible outcome population and were not treated as intervention-effect evidence (60, 75). This distinction preserves the prespecified crisis-exposure and MHPSS eligibility boundaries.

Several limitations constrain the conclusions. The protocol was not prospectively registered. The included evidence was clinically and methodologically heterogeneous, and the number of robust randomized trials was limited, especially in post-earthquake and community-based humanitarian settings. Some studies had small samples, short follow-up, incomplete reporting of adherence or facilitator training, and unclear intervention fidelity. Controlled and uncontrolled evidence required separation, and outcomes such as suicidal ideation, community resilience, social connectedness, and life skills were represented by few or non-comparable studies. Small outcome sets also limited the power of funnel plots and regression-based publication-bias tests; the absence of a strong signal cannot be interpreted as absence of bias. Finally, the broad crisis scope—including disaster, conflict, displacement, refugee settings, and pandemic restrictions—supports a cross-context MHPSS perspective but limits direct generalization to any single disaster type.

Future studies should preregister protocols, use adequately powered randomized or well-controlled comparative designs, report allocation, attrition, adherence, intervention fidelity, adverse events, and safeguarding, and include longer follow-up. Core outcome sets should distinguish clinical symptoms from psychosocial and community constructs. Component and mediation analyses are needed to determine whether physical exertion, routine, group belonging, facilitator relationships, protection content, or life-skills modules drive change. Equity analyses should examine age, gender, disability, socioeconomic position, displacement status, and cultural context.

### 4.6 Conclusion

Sport-, play-, exercise-, movement-, physical-education-, yoga/body-awareness-, and sport-for-protection-based interventions may contribute to post-disaster and humanitarian mental health and psychosocial recovery. The pooled findings indicate favorable small-to-moderate average effects on PTSD, depression, and anxiety, while the narrative evidence identifies possible contributions to routine, regulation, belonging, social connection, life skills, and community cohesion. Confidence is moderated by heterogeneity, risk of bias, small outcome sets, and variation in programme content and context. Sport-based interventions should therefore be implemented as complementary, safeguarded, culturally adapted components of multi-layered MHPSS systems—not as replacements for trauma-focused treatment, clinical risk management, child protection, or specialized mental health care.

## Data Availability

All data analyzed in this study were derived from previously published studies and publicly available sources. No new primary data were generated for this systematic review and meta-analysis.
